# Surgical outcomes and observation in exotropia cerebral palsy children with cortical visual impairment

**DOI:** 10.1186/s12886-022-02581-x

**Published:** 2022-09-08

**Authors:** Haiyun Ye, Qingyu Liu, Qijia Zhan, Yidan Zhang, Xiaodong Du, Xiaoxiao Zhang, Yue Di, Tong Qiao

**Affiliations:** 1 Department of Ophthalmology, Shanghai Children’s Hospital, Shanghai Jiao Tong University, Shanghai, 200062 China; 2 Department of Neurosurgery, Shanghai Children’s Hospital, Shanghai Jiao Tong University, Shanghai, 200062 China

**Keywords:** Exotropia, Cerebral visual impairment, Cerebral palsy, Contrast sensitivity testing, Functional vision assessment

## Abstract

**Purpose:**

Cortical visual impairment (CVI) is the common cause of pediatric visual impairment in cerebral palsy (CP) while exotropia is the most common strabismus associated with CP. We aim to observe the strabismic surgery outcomes in pediatric patients with CP and CVI.

**Method:**

Our medical records were collected from pediatric patients treated in our hospital from May 1, 2017 to Jan 1, 2022. With normal intelligence assessment and diagnosis of exotropia in children with CP and CVI, microsurgeries were performed under intravenous combined inhalation anesthesia. The strabismus was examined by the prism test under best vision correction and the contrast sensitivity testing (CST) was measured at five levels of spatial frequencies.

**Result:**

A total of 38 exotropia patients with CP and CVI were identified and included for analysis during the study period with age ranged from 5 to 12 years (mean 8.45 years) and mean follow up duration was 8.7 months (6–42 months). After bilateral lateral rectus recession (with/without medial rectus resection or inferior oblique transposition), the exotropia amount of participants were obviously revealed from − 30 ~ − 140 (median, IQR: − 50, 40) prism diopters (PD) preoperatively to 0 ~ − 15 (0, 5) PD postoperatively. Statistically significantly improvements were observed at all levels of spatial frequency on CST postoperatively, especially at high spatial frequency areas (*p* < 0.05).

**Conclusion:**

Our results demonstrated that the effect of strabismus surgery on exotropia in children with CP and CVI were stable and monocular contrast sensitivity post- operation increased significantly at all spatial frequencies levels.

The brain is vulnerable to various assaults that lead to cerebral lesions in developing period (before, during, or after birth). Cerebral palsy (CP) is one of the nonprogressive brain disorders that affects the immature brain. All CP children have difficulties in controlling motor muscles and can be classified as spastic, ataxic, athetoid, or atonic type according to motor abnormalities [1]. The majority of CP children may suffer from more than one concurrent disability (sensory, motor, mental, learning, etc). Strabismus, especially exotropia, is one of the most common ocular morbidities associated with CP, with higher rates than those detected in general population [2, 3]. Moreover, in pediatric CP patients, visual acuity problems (such as strabismus, hyperopia, myopia and amblyopia) lead to the crowding phenomenon and to visual maturation delay [4]. Cortical visual impairment (CVI), which is the result of post geniculate visual pathways damage that leads to deficits in visual function, is the most common cause of pediatric visual impairment in developed and certain developing countries according to previous researches [5]. In our study, we aim to observe the exotropia in children with CP and CVI pre- and post- strabismic surgery outcomes.

## Methods

This study was approved by the Institutional Review Board of Shanghai Children’s Hospital. We collected the medical records of exotropic pediatric patients treated in ophthalmic outpatient department in our hospital between May 1, 2017 and Jan 1, 2022, who had incurred an ICD-9 code for CVI with CP were reviewed. CVI was diagnosed visual impairment clinically with no ocular or anterior visual pathway pathology sufficient to explain the level of impairment. Age at first ophthalmologic examination ranged from 2 to 12 years, considering the patients’ cooperation on strabismus examination and CST as well as the guardian’s intention, all children under 4 years old were scheduled for rehabilitation training before ophthalmology surgery. Some of them followed up for nearly 5 years until strabismus operation. Based on the normal intelligence assessment, comprehensive ophthalmologic examinations were performed on exotropia children with CP and CVI enrolled including visual acuity evaluation, ocular motility, stereoacuity (Randot stereoacuity test), binocular vision, cycloplegic refraction by one author (TQ). Slit lamp and fundus examination were carried to exclude corneal erosions, lens opacity, optic disc or fundus disease. Orthoptic patch was applied before angle measurement to ensure the examination stability of alternate prism and cover test. The visual acuity with cycloplegic refraction was tested by Snellen test at a fixed point of 5 m. Exclusion criteria were as follows: no diagnosis of CVI, no diagnosis of strabismus (exotropia), esotropia with CVI, less than 4 years old at surgery (sufficient vision improvement and binocular cognitive development), abnormal intelligence assessment, inadequate documentation records, low functioning CP/CVI patients [6]. Ocular deviation was examined using traditional prism inspection (more than ten times) by Dr. Qiao. The examinations were repeated five to ten times (not in 1 day) and the average value of two times with high compatibility was taken. Exotropia with CP and CVI children were performed microsurgery of bilateral lateral rectus muscle recession (BLR) (with/without medical rectus resection, inferior oblique transposition) under intravenous combined inhalation anesthesia. The following information and data were recorded before and after surgery: underlying cause of CVI, sex, strabismus type and size as well as strabismic deviation degree (minus “-” denotes esotropia and plus “+” means exotropia). Contrast sensitivity testing (CST) was measured at 1.5, 3, 6, 12, and 18 cycles/ degree (cpd) spatial frequencies by using the DOBOSO 3D vision system (Guangzhou Shijing Medical Software Co., Ltd). Each participant underwent monocular CST with best refractive correction under luminance conditions of 85 cd/m^2^. Spatial frequencies were presented randomly in the range of zero contrast to the grating contrast that could just be detected by subjects. Each grating was tested three times and the mean measurements were recorded. Statistical analysis was performed using log (CST) and the paired t test on pre- and post-operation variables. The patients were arranged 1 day, 1 week, 1 month, 3 months, 6 months and 1 year after surgical treatment to follow-up. Statistical significance was defined as *p* < 0.05, using 2-sided alternative hypotheses. Data were analyzed using SPSS 23.

## Result

**Fig. 1:**
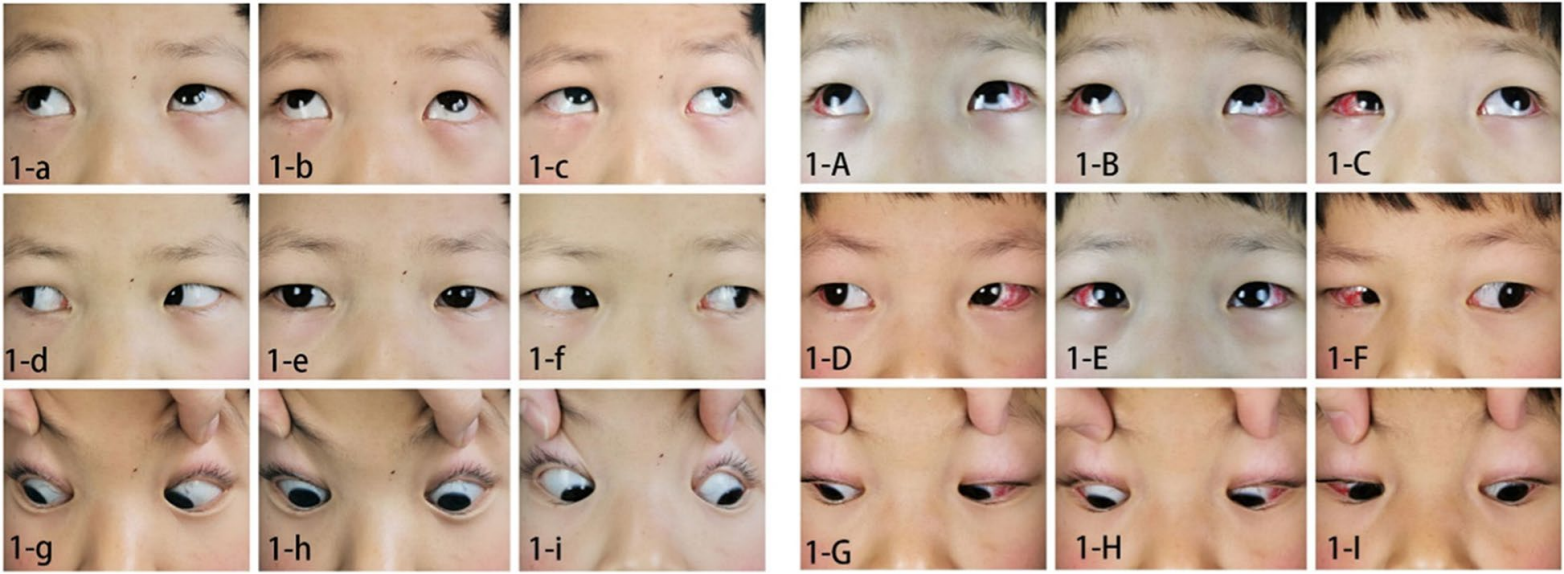
Nine gaze positions of an exotropia CVI children with CP: 1-a ~ 1-i showed exotropia before BLR recession; 1-A ~ 1-I showed: exotropia was obviously improved 1 day post-operatively. (CVI: cerebral visual impairment; CP: cerebral palsy; BLR: bilateral lateral rectus)

A total of 38 exotropia children with CP and CVI were identified and included for analysis during the study period (23 male and 15 female) ranged from 5 to 12 (mean 8.45) years and mean follow up duration was 8.7 months (range 6–42 months). Of the 38 participants, 25 had constant exotropia and 13 had intermittent exotropia. Best corrected visual acuity ranged from 10/20 to 20/20. The amount of exotropia ranged from − 30 ~ − 140 prism diopters (PD) (median, interquartile range (IQR): − 50, 40) preoperatively vs 0 ~ −15PD (0, 5) postoperatively (Table 1). Exotropia of all the exotropia children with CP and CVI were obviously revealed after BLR recession (with/without medical rectus resection), including twelve patients showed inferior oblique overaction and oblique muscle transposition surgery was performed at the same time (Fig. 1). Besides, statistically significantly improvements (*p* < 0.05) were observed at all levels of spatial frequency in monocular CST in both eyes of CVI children postoperatively (follow-up range 3 ~ 12 months, mean: 6.16 ± 2.43), especially at high CST spatial frequency areas (6, 8, 12 cpd) (Table 2).

**Table 1:** Pre- and post-operative data and evaluations

Serial Number	Age (years)	Sex	Follow-up (months)	Pre- PCT (PD, △)	Post- PCT (PD, △)	Surgery performed (BLR, mm)	Surgery performed (MRR, mm)	Surgery performed (with IO transposition, Y/N)	CST follow-up duration (months)	BCVA OD	BCVA OS	Refraction OD	Refraction OS
**1**	6	M	31	−50	−5	10		N	3	0.9	0.9	+ 2.00DS –0.75DC×15	+ 2.50DS –0.50DC×10
**2**	8	M	33	− 60	− 5	9	4	N	3	0.7	0.9	− 4.00DS - 1.00DC×45	− 2.50DS - 2.50DC×145
**3**	6	F	10	− 50	−5	10		N	3	0.8	0.8	+ 2.75DS + 0.50DC×144	+ 2.75DS –1.00DC×15
**4**	10	M	10	−90	0	10	4.5	Y	3	0.9	0.9	+ 2.00DS	+ 2.25DS –0.25DC×70
**5**	8	M	9	−40	0	9		Y	6	0.8	0.8	+ 1.50DS –2.50DC×175	+ 2.00DS –2.50DC×5
**6**	8	F	41	−30	−5	7.5		Y	3	0.7	0.8	+ 0.50DS –1.50*12	+ 0.50DS
**7**	6	M	41	−140	−5	9	7.5	N	9	0.9	0.9	+ 2.75DS	+ 2.75DS
**8**	11	M	42	−40	0	9		Y	3	0.8	0.9	+ 2.75DS –0.50DC×15	+ 1.50DS –0.75DC×180
**9**	9	M	42	−70	0	9	5	N	3	0.8	0.9	− 0.50DC×10	+ 0.50DS –0.50DC×175
**10**	9	F	42	−40	0	9		N	6	0.8	0.8	+ 4.25DS –1.00DC×160	+ 5.00DS –1.50DC×20
**11**	7	M	6	−50	−5	10		N	6	1.0	1.0	− 0.25DS - 3.00DC×5	− 0.25DC×4
**12**	8	M	6	−50	−10	10		Y	6	0.9	0.8	+ 3.50DS –1.50DC×160	+ 3.00DS –0.75DC×15
**13**	7	F	31	−60	5	9	4	N	6	0.9	0.8	+ 1.25DS	+ 1.25DS
**14**	6	M	30	−60	−10	9	4	Y	3	0.8	0.8	+ 0.50DS –0.25DC×170	+ 0.75DS –0.50DC×15
**15**	9	F	33	−90	−10	10	4.5	Y	6	1.0	1.0	− 1.25DS - 0.50DC×170	− 1.50DS - 0.75DC×5
**16**	11	F	25	−40	0	9		N	9	0.9	0.8	+ 1.00DS –1.00DC×5	+ 0.50DS –0.75DC×5
**17**	12	M	12	−40	0	9		N	6	0.7	0.8	− 1.75DS - 0.75DC×15	− 0.50DS -1.25DC×180
**18**	6	F	19	− 100	−5	10	6	N	3	0.6	0.8	− 0.75DS - 1.75DC×175	− 2.25DS - 1.25DC×180
**19**	10	M	10	−40	0	9		N	9	0.8	0.7	− 1.75DS	+ 3.25DS
**20**	8	F	40	−80	−5	9.5	5.5	Y	12	0.9	0.8	− 2.50DS	− 0.75DC×145
**21**	10	F	9	−40	0	9		Y	6	0.7	0.8	+ 2.50DS –2.25DC×15	+ 0.75DS –1.25DC×180
**22**	11	M	16	−80	−10	9.5	5.5	N	3	0.8	0.6	− 1.75DC×30	+ 1.25DS –1.75DC×180
**23**	8	M	41	−50	0	10		N	6	0.9	0.8	− 0.75DS - 1.75DC×175	− 2.25DS - 1.25DC×5
**24**	7	F	33	−60	0	9	4	N	6	1.0	0.8	− 1.00DS	− 0.50DS
**25**	10	M	41	− 50	5	10		Y	6	0.8	0.9	+ 0.75DS –0.50DC×5	+ 0.75DS –0.75DC×5
**26**	9	M	27	−90	−5	10	4.5	N	6	0.8	1.0	+ 3.75DS –0.75DC×180	+ 3.25DS –1.00DC×180
**27**	9	F	42	−40	0	9		N	9	0.9	0.8	+ 2.25DS –1.00DC×175	+ 3.75DS –2.25DC×175
**28**	8	M	18	−40	0	9		N	3	1.0	1.0	+ 0.50DS –0.75DC×170	+ 0.25DS –0.75DC×180
**29**	6	F	42	−70	5	9	5	N	6	0.9	0.9	+ 0.25DS –0.50DC×155	− 0.50DC×155
**30**	12	F	20	−40	0	9		N	9	0.7	0.8	+ 2.25DS –0.50DC×25	+ 1.00DS
**31**	11	F	42	−80	−5	9.5	5.5	N	9	0.9	1.0	− 3.00DS - 0.75DC×10	− 2.75DS
**32**	7	M	27	−40	5	9		N	3	0.8	0.7	− 2.25DS + 1.00DC×180	− 1.75DS
**33**	8	M	6	−80	0	9.5	5.5	N	6	0.7	0.9	− 1.00DS − 0.50DC×175	- 0.50DC×175
**34**	9	M	9	−50	0	10		Y	6	0.8	0.8	+ 0.75DS –0.50DC×180	+ 0.50DS –1.25DC×180
**35**	10	M	6	−80	10	9.5	5.5	N	6	0.9	0.8	+ 1.00DS –0.75DC× 175	+ 1.00DS –0.75DC×175
**36**	10	F	9	−60	0	9	4	N	3	1.0	1.0	+ 0.50DS –0.75×170	+ 0.25DS –0.75×180
**37**	6	M	28	−80	−5	9.5	5.5	Y	9	0.9	0.8	+ 0.75DS –0.75DC×155	+ 1.00DS –0.75DC×10
**38**	8	M	42	−50	0	10		N	3	0.7	1.0	+ 0.50DS –0.75DC× 165	− 0.50DS - 0.5DC× 20

*PCT* Prism Cover Test, *BLR* bilateral lateral rectus recession, *MRR* medial rectus resection, *IO* inferior oblique, *Y/N* Yes (with)/No (without), *CST* contrast sensitivity testing, *BCVA* best corrected visual acuity. “-”: denotes esotropia

**Table 2:** All levels of spatial frequency on monocular CST (log CST) in both eyes of exotropia CP with CVI children significantly improved postoperatively, especially at high CST spatial frequency areas (6, 8, 12 cycles/ degree)

cpd	OD					OS				
	1.5	3	6	12	18	1.5	3	6	12	18
**Pre-surgery**	**1.90 ± 0.10**	**2.14 ± 0.12**	**2.12 ± 0.06**	**1.83 ± 0.12**	**1.52 ± 0.14**	**1.88 ± 0.10**	**2.07 ± 0.11**	**2.10 ± 0.06**	**1.84 ± 0.11**	**1.54 ± 0.14**
**Post-surgery**	**1.97 ± 0.06**	**2.21 ± 0.03**	**2.20 ± 0.04**	**2.04 ± 0.04**	**1.76 ± 0.06**	**1.97 ± 0.06**	**2.21 ± 0.03**	**2.21 ± 0.03**	**2.05 ± 0.03**	**1.76 ± 0.05**
**t**	**7.52**	**3.68**	**9.99**	**10.10**	**10.78**	**8.53**	**8.37**	**11.96**	**10.84**	**10.93**
**p**	0.00*	0.00*	0.00*	0.00*	0.00*	0.00*	0.00*	0.00*	0.00*	0.00*

*CST* contrast sensitivity testing, *CP* cerebral palsy, *CVI* cerebral visual impairment, *cpd* cycles/ degree, *: statistically significant difference (*p* < 0.05)

## Discussion

Complex activity of cerebral visual processing involved a large part of the central nervous system [7, 8]. CVI is a disorder caused by damage of the retro geniculate visual pathways, particularly loss of central visual acuity and visual field defects, and is considered the most important cause of visual dysfunction in children in developed countries [5]. CVI were diagnosed by neurologist of MDT team in our hospital. In all participants, magnetic resonance imaging (MRI) was taken, in which periventricular leukomalacia can be found. CVI underlying cause were pre-, peri-, or postnatal and may involving but not limited to seizures, hydrocephalus, perinatal stroke, congenital central nervous system malformations, or greater than one insult [9]. CVI children may present with unique characteristics and require personal treatment strategies according to ocular pathology. Pediatric CVI ophthalmological abnormalities (decreased visual acuity, visual field defects, oculomotor problems, cognitive visual dysfunction) were known to differ according to CP types [10, 11]. Ocular abnormalities including refractive errors (myopia, hyperopia and astigmatism), strabismus, nystagmus, and amblyopia as well as CVI were observed in 50–90% of the patients with CP [2, 10, 11]. The rate of coexistent strabismus has been reported to be as high as 73% in patients with CVI, yet no clear guideline existed for management on their strabismus [6, 12, 13]. Researchers had concluded that exotropia with CP did not differ from exotropia without CP in surgical responses, surgical success rates, cumulative probabilities of surgical success or recurrence after BLR recession. According to the Parks’ formula, Lee et al. augmented BLR recession for basic intermittent exotropia and showed that increasing the amount of recession by 1.5 mm to 2.5 mm per eye improved success rate compared to that of the conventional degree of recession [14]. However, they did not include the patient with CP. Dae et al. modified that increasing the surgical amount of BLR recession by 1.0 mm per eye may be safe and efficient in exotropia with CP [2]. Consistent with Dae, we increased BLR recession amount of 1.0 mm and the surgical results were satisfactory and stable. The personal dose design depends not only on the formula but also the muscle conditions (with/without abnormal extraocular muscle development, muscle tension differences et al) in each patient. With a tendency for CVI patients’ visual acuity improvement with time and neuroplasticity, strabismus remission or persistence will affect whether the strabismus be treated surgically. In some observations, spontaneous resolution of strabismus occured with vision improvement, and no surgery was needed [15]. Occasionally, for psychosocial consideration, parents of strabismus children with CVI may elect ameliorative surgery [15]. Prudently, before the procedure, we fully communicated with the children’s guardians about the purpose of surgery and clarified their surgical requirements, so as to avoid any misunderstanding of the significance of surgery.

Although the intellectual impairment associated with CP in children was recognized, the CVI participants we enrolled were normal in terms of intelligence assessment, understanding and cooperation on examination. Contrast sensitivity was an important portion in visual function, nevertheless, it had not been fully evaluated in pediatric patients with strabismus and CVI. Considering the cooperation of these special subjects, only high functioning CP patients were included in our research. Previous researchers found that, compared with age matched controls, insufficient contrast sensitivity were observed in CVI children [16]. Compared with preoperative data, our results demonstrated that strabismus children with CVI had monocular contrast sensitivity increase at all levels of spatial frequency and significantly at high CST spatial frequency areas, which complemented previous studies.

However, there were some limitations in our study. The improvement of monocular CST was indeed gratified, but the visual quality and patient adaptation of binocular simultaneous vision had not been tracked. Through this study, we believed that there were many factors influenced the effect of strabismus surgery for exotropia in children with CP and CVI, including the patient’s basic condition, the degree of bio-family-social rehabilitation training, and the surgical design. As patients suffered problems in neurology, ophthalmology, rehabilitation and other aspects, it had certain requirements on the patients’ family economic conditions, parental awareness and ability. The majority of our study enrollments’ family had long experience in seeking treatment, therefore, they may have certain medical knowledge and good compliance. Many researchers reported that exotropia was far more common in the Asian population, even in patients with CP [3]. Since the incidence rate, cognition and coordination ability, we just focused and analyzed the exotropia CVI patients with CP. Moreover, it was generally believed that exotropia usually did not interfere with the development of vision or visual function. The subjects in our study were special and the CST examination was relatively poor. We analyzed that this may be related to the general condition: most of the subjects had a history of hypoxia or even asphyxia. Besides, potential factors may affect surgical response such as A or V pattern, amblyopia and stereopsis, which could not be fully evaluated. We hypothesized the remodeling of eye position by strabismus surgery may be beneficial to the visual pathway in brain. However, there was no relevant mechanism research to support this idea at present. We hope to take our result as an opportunity to provide idea for future mechanism research in exotropia children with CP and CVI. Based on current knowledge, we speculated that the recognition of the CVI strabismus children may be remolded after the surgery and early identification of CVI is important not only for families but also may help treatment strategy. We will further study the relation between strabismus and the CVI recognition in future.

## Data Availability

The data used to support the findings and the video of the operation of this study were available from the corresponding author upon request.

## References

[1] Bax MC (1964). Terminology and classification of cerebral palsy. Dev Med Child Neurol.

[2] Ma DJ, Yang HK, Hwang JM (2017). Surgical responses and outcomes of bilateral lateral rectus recession in exotropia with cerebral palsy. Acta Ophthalmol.

[3] Woo SJ, Ahn J, Park MS, Lee KM, Gwon DK, Hwang JM (2011). Ocular findings in cerebral palsy patients undergoing orthopedic surgery. Optom Vis Sci.

[4] Kozeis N, Panos GD, Zafeiriou DI, de Gottrau P, Gatzioufas Z (2015). Comparative study of refractive errors, strabismus, microsaccades, and visual perception between preterm and full-term children with infantile cerebral palsy. J Child Neurol.

[5] Pehere NK, Jacob N (2019). Understanding low functioning cerebral visual impairment: an Indian context. Indian J Ophthalmol.

[6] Binder NR, Kruglyakova J, Borchert MS (2016). Strabismus in patients with cortical visual impairment: outcomes of surgery and observations of spontaneous resolution. J AAPOS.

[7] Edmond JC, Foroozan R (2006). Cortical visual impairment in children. Curr Opin Ophthalmol.

[8] Fazzi E, Signorini SG, La Piana R, Bertone C, Misefari W, Galli J (2012). Neuro-ophthalmological disorders in cerebral palsy: ophthalmological, oculomotor, and visual aspects. Dev Med Child Neurol.

[9] Philip SS, Dutton GN (2014). Identifying and characterising cerebral visual impairment in children: a review. Clin Exp Optom.

[10] Ghasia F, Brunstrom J, Gordon M, Tychsen L (2008). Frequency and severity of visual sensory and motor deficits in children with cerebral palsy: gross motor function classification scale. Invest Ophthalmol Vis Sci.

[11] Saunders KJ, Little JA, McClelland JF, Jackson AJ (2010). Profile of refractive errors in cerebral palsy: impact of severity of motor impairment (GMFCS) and CP subtype on refractive outcome. Invest Ophthalmol Vis Sci.

[12] Huo R, Burden SK, Hoyt CS, Good WV (1999). Chronic cortical visual impairment in children: aetiology, prognosis, and associated neurological deficits. Br J Ophthalmol.

[13] Fazzi E, Signorini SG, Bova SM, La Piana R, Ondei P, Bertone C (2007). Spectrum of visual disorders in children with cerebral visual impairment. J Child Neurol.

[14] Lee SY, Hyun Kim J, Thacker NM (2007). Augmented bilateral lateral rectus recessions in basic intermittent exotropia. J AAPOS.

[15] Collins ML (2014). Strabismus in cerebral palsy: when and why to operate. Am Orthopt J.

[16] Good WV, Hou C, Norcia AM (2012). Spatial contrast sensitivity vision loss in children with cortical visual impairment. Invest Ophthalmol Vis Sci.

